# Abstract of Proceedings of the Buffalo Medical Association

**Published:** 1877-08

**Authors:** E. N. Brush

**Affiliations:** Secretary


ART. II.—*Abstract of the Proceedings of the Buffalo Medical Association, Tuesday Evening, July* 3d, 1877. E. N. BRUSH, M. D., Secretary.

In the absence of the President, the meeting was called to order by the Vice-President, Dr. Thomas Lothrop. There were present Drs. Cronyn, Gould, Hopkins, Howe, Fowler, Abbot, Rochester, J. F. Miner and Brush.

On motion of DR. HOWE the reading of the minutes of the last meeting were dispensed with.

DR. H. R. HOPKINS read the following paper on "Spontaneous Generation."

*Mr. President and Gentlemen:*

In inviting your attention to some considerations upon Spontaneous Generation, I am aware that I am departing somewhat from the practice of your former essayists.

The reasons for this departure are two-fold—those relating to myself, and those pertaining to the subject.

Without doubt we are all of one mind, that study to be either enjoyable or profitable must be by subject; that disjointed, discursive reading is injurious in direct proportion to the extent it is practiced, that such reading fills the mind with chaotic facts, facts which cannot be used in constructive thought, and which occupy the mind to the exclusion of those facts, which, from their adaptability to the wants of the mind, we have come to designate as practical facts.

Then again, the most profitable mental work is that which suggests itself, the rule that the willing hands do the most work, being just as applicable to the realm of thought as to the cotton-field.

Upon receiving the compliment of an invitation to prepare a paper for this evening's entertainment, the operation of the previous mentioned laws led me to select the subject of Spontaneous Generation, partly because it is included in the subject which has for some time engrossed my leisure moments, and partly because of the intrinsic interest of the subject itself; and again for the reason that never, since this subject has occupied the thoughts of scientific men, has the interest been so intense as now, for the reason that the final settlement of the question seems close at hand.

When the so-called doctrine of spontaneous generation originated, cannot be determined, but there is abundant evidence to show that down to the 17th century it was a universally conceded doctrine. The reasons for the general acceptance of this law you will at once see, when you consider the manner in which men were wont to observe natural phenomena, and the loose inferences they were accustomed to draw from such phenomena.

When men saw that most animal, and many vegetable substances upon undergoing decomposition produced large numbers and many varieties of living forms, and that even pure water on becoming stagnant, was found to be teeming with life, what was more unreasonable than the inference that the living objects came directly from dead matter. At any rate, this belief was held by all classes, the most learned and the unlearned.

In the year 1668 Francesco Redi, an Italian, published a book which was destined to shape the thought of the world for generations. This book contained an account of some experiments which he had made, and which led him to deny the truth of the doctrine of spontaneous generation, a doctrine honored with universal and immemorial acceptance, and to announce in its place the doctrine "*Omne vivum ex ovo.*" To physicians who remember that the science of medicine owes its birth, or its regeneration, to the labors of those noble sons of Italy, Sylvius Vesalius, Fallopius and others, and to the students of history who recall that what Germany is to the thought of the 19th century, that Italy was to the learning of the 15th and 16th centuries, it will seem fit and proper that it was left for an Italian to correct the error in observation and the error in inference of the ages which had preceeded him regarding the origin of living beings. And it is worthy of notice that Redi's success was owing to the fact that he set himself to study and observe nature and natural phenomena, rather than allow himself to speculate as to the cause of supposed phenomena. As you might suppose, a terrible storm was raised over the new doctrine, and men then did as men do now, they looked at the new doctrine and at its propounder, and then attacked whichever they preferred. Fortunately for Redi, his strength was not in the ingenuity of his deductions, but in the originality of his facts, and his opponents invariably retired from the contest worsted, and before the close of the century the doctrine of Biogenesis, that life comes through the agency of previous living matter, was without opposition. Before the close of the 18th century new features presented themselves in the case. By this time that aggressive habit of mind which in the 16th and 17th centuries was so peculiar to Italy, had spread throughout Europe, and the old questions were being studied by new men. Two naturalists, Needham of England and Buffon of France, took up the study of this subject and found that they could not agree with the hypothesis of Redi, for the following reasons: Needham, writing in 1850, says in substance the theory of Biogenesis claims that the organic life appearing in a decomposing vegetable infusion, is the result of the development of the infusosial germs which that iafusion contains. We know that heat kills all living germs. I accordingly boil this bottle of vegetable infusion, and when hot cork it tight and cover the cork with mastic and set it aside; there is now no living germs here and if the theory of Biogenesis is true there will be no life here; but in fact after waiting for a few days the bottle swarms with living forms. These experiments were verified by other observers, and inasmuch as they seemed to flatly contradict the tradition "*Omne vivum ex ovo*," the rival doctrine of spontaneous generation resumed its former place. Before many years the scene again shifts, and this time back to the place of its birth in Italy. The Abbe Spallanzani criticised the work of Needham and Buffon, that their facts did not warrant their conclusions, that the degree of heat used was not sufficient to kill the germs, and that the corks and mastic did not effectually keep out the air and the germs which it contained. Spallanzani boiled the infusions for a quarter of an hour, and then closed the bottles, by hermetically sealing their necks while boiling, and then found that life failed to appear.

On the face of these facts the supporters of spontaneous generation were silent, and again the pendulum of belief run to the side of Biogenesis.

The close of the 18th century witnessed the introduction of a new element into the controversy, and one of so much importance that the whole ground must be traversed again. About this time chemists and physiologists were becoming acquainted with the influence of oxygen upon vital phenomena, respiration and nutrition, and men said, is it not oxygen that the infusions of Spallanzani want, to make them fruitful; and finally came to demand that the crucial experiment must give all the favorable conditions for the support of life, at the same time that all existing life was excluded. Since the year 1836 the most able naturalists, chemists, physicists and biologists, have been at work upon this problem, and there are yet two parties, each of which is positive that the truth is with them.

In mentioning the champions of Biogenesis, we must place at the head Pasteur, and then can cite such names as Helmboltz, Tyndall, Boussingault, Dumas, Roberts and Dallinger; and in the list of the advocates of spontaneous generation or Abiogenesis, Bastian and Burdon-Sanderson of England, Cohn of Breslau, and Huitzinga of Groningen. Says a recent writer: "By a long course of patient work, controlled by eager criticism, the methods of procedure have been gradually perfected and simplified. The opposing ranks have closed, as it would seem, for their decisive struggle. The champions on either side have pressed to the front and we may reasonably hope before long to see the final upshot of a controversy as old as scientific thought." I will not weary you with the details of the various experiments which have been made over and over again by various students of this subject, but will briefly indicate where an experiment has succeeded in establishing a decisive point. Helmholtz, from his studies of the yeast plant and its relations to fermentation, established the fact that, the substance which excites fermentation and putrefaction, and at the same time gives rise to living forms in a fermentable or putrescible fluid, is something which cannot pass through an animal membrane; therefore is not a gas or a diffusible liquid, therefore it is either a colloid or it is matter divided into very minute solid particles.

Posteur demonstrated that these particles of solid matter could be sifted from the air by a plug of cotton wool. He proved their existence in the wool by sowing them in a proper soil and seeing them grow, and he again proved their solid nature by connecting his flasks with the air by a bent tube, in which the germs would be obliged to ascend in order to reach the fluid, and under these conditions found the fluids invariably sterile. Tyndall has shown that by means of a beam of electric light, you can show the presence of the infusorial germs in the air, and that when a portion of confined air has been allowed to settle until the passage of the beam shows no track, vegetable or animal infusions can be opened in it and remain barren of life; while Dallinger has proven the most suggestive fact that wherever a putrid infusion dries up, there will be found a powdery mass containing spores, which every breath of air will diffuse far and wide, and that some of these spores are so minute as to require two days to fall a few inches in a perfectly still atmosphere. So that the distance to which they could be carried, and to which they could spread contagion is practically unlimited.

On the other hand, Bastian has demonstrated and Cohn, Sanderson and Huitzinga have confirmed that the needful conditions for the development of life in boiled organic infusions are the neutrality or slight alkalinity of the fluid, and its maintainance at a temperature of 115° to 120° F. Also that a turnip infusion with cheese dust added will produce life, after having been boiled and protected from contact with the atmosphere or anything it may contain. Huitzinga has ascertained the like fact when soluble peptons was substituted for cheese dust, and no counter experiments on this point have been made public.

The like fact with other purely liquid infusions has been alleged by Bastian, acknowledged by Sanderson, and confirmed by Huitzinga and Cohn.

Bastian's most telling experiment which he communicated to the Royal Society on June 15th, 1876, and subsequently to the French Academy, is as follows: He places in a retort a given quantity of urine of a known acidity, then he places in a small fine tube enough liquor potassa to not quite neutralize the acid of the urine; this tube he boils from ten to fifteen minutes and seals it when boiling. The tube is then dropped into the retort of urine, and the whole is boiled for some moments and hermetrically sealed while boiling; a number of retorts thus prepared are placed in a bath of 122° F. and all but one or two are shaken so as to break the potash tube and thus neutralize the acidity of the urine. In almost every case, the retorts thus prepared show life in a few days, while those in which the inclosed potash tubes are unbroken, invariably remain sterile. The receipt of these experiments threw the French Academy into quite an excitement, and Messrs. Boussingault, Milne Edwards, and Dumas were appointed a commission to overlook Dr. Bastian's experiments.

It is also suggested that a similar commission be appointed by the Royal Society to report upon the contra experiments to be made by Posteur or Tyndall.

Let us hope that the truth which has so long eluded the search the most patient and persistent investigators will soon be brought to light. Against the general trustworthiness of Dr. Bastian's views, it is urged, that he is influenced by his philosophy, namely. "That continuity in nature is the grand outcome of all modern research; but if you are to have this in a sense wide enough to include the organic world, you must have spontaneous generation. Give up this, and continuous evolution is impossible; therefore Abiogenesis must be a great truth." The importance of this doctrine to the hypothesis of evolution is indicated by Tyndall's clasic reference to the "power and potency of matter," and the drift of the advanced thought is shown by the following words of Professor Huxley: "But I must guard myself against the supposition that I intend to suggest, that no such thing as Abiogenesis has taken place in the past, or ever will take place in the future. With organic chemistry, molecular physics, and physiology yet in their infancy, and every day making prodigious strides, I think it would be the height of presumption for any man to say that the conditions, under which matter assumes the properties we call 'vital,' may not some day be artificially brought together. All I feel justified in affirming is, that I see no reason for believing that the feat has been performed yet. And looking back through the prodigious vista of the past, I find no record of the commencement of life, and therefore I am devoid of any means of forming a definite conclusion, as to the conditions of its appearance. Belief in the scientific sense of the word, is a serious matter, and needs strong foundations. To say, therefore, in the admitted absence of evidence, that I have any belief as to the mode in which the existing forms of life have originated, would be using words in a wrong sense.

But expectation is permissible when belief is not, and if it were given me to look beyond the abyss of geologically receorded time to the still more remote period, when the earth was passing through physical and chemical conditions which it can no more see again than a man can recall his infancy, I should expect to be a witness of the evolution of living protoplasm from non-living matter.

I should expect to see it appear under forms of great simplicity, endowed like existing fungi, with the power of determining the formation of new protoplasm from such matters as ammonium carbonates, oxalates and tartrates alkaline, and earthy phosphates and water, without the aid of light. That is the expectation to which analogical reasoning leads me, but I beg you once more to recollect that I have no right to call my opinion anything but an act of philosophical faith."

Dr. Abbott in speaking on the paper, expressed his sorrow that no more were present to hear it. He then referred to the experiments of Tyndall as narrated in an article by that gentleman in the *Popular Science Monthly*, entitled, "A Combat with an Infective Atmosphere."

He said that in the experiments made by Tyndall, in the fall of 1875, no life appeared in animal and vegetable infusions which he made and kept in his "chambers" or boxes, free from exposure to air from the outside, but upon repeating these experiments in 1876, in October, November and December, he was unable to keep the infusions free from life. After repeated trials he moved the apparatus and commenced a new series of experiments in Kew Gardens. Here the results were perfect, the infusions remained perfectly clear and free from life. In seeking for an explanation of this he found that his laboratory where the experiments were first tried, had in it some old hay, the germs of which had, no doubt contaminated the atmosphere. These germs not being destroyed by the short boiling which was necessary to kill the others, had prevented the success of his experiments.

Dr. Howe said he was glad Dr. Hopkins had brought this subject forward; it is one of practical interest to physicians. Tyndall's article, which was termed a combat with an infective atmosphere, contained some suggestive facts for physicians in the causation of contagious diseases.

Dr. Cronyn said that to surgeons, this matter of disease-germs, bacteria, etc., was of great interest. They were constantly bringing wounds into contact with the air, which might be vitiated with these germs. Prof. Lister has evidently anticipated the result of the discussion in reference to spontaneous generation by the employment of his antiseptic method to destroy the germs, which he considers necessary to create putrefactive changes in wounds. He referred to a paper read by Dr. Grant, before the Canadian Medical Association, on this subject and concluded by suggesting that cleanliness was probably the main essential in the treatment of wounds, and asking whether the old "Friar's Balsam" which some one had recently revived, was not as equally efficacious as Lister's method.

Dr. Rochester said he had the pleasure of listening to Prof. Lister as chairman of the section on Surgery in the International Medical Congress, deliver an address on antiseptic surgery. During this address, and during Dr. Hopkins' paper, one thought had struck him. If a brief exposure to boiling water will not destroy these germs, what real power can reside in a spray of carbolized material to prevent their action.

Dr. Miner said that he had but one suggestion to make on this subject. Surgeons were constantly reporting new methods of dressing and treating wounds, and claiming wonderful results therefor. The fact was, wounds did equally well under almost any dressing. One man used the open method of treating a stump, another the method of Lister, a third, hermetically so to speak, sealed up the dressings and made them permanent, while still another immersed the member in hot water. Under different circumstances all were equally successful.

Dr. Brush said that he had listened with a great deal of interest to Dr. Lister's lengthy address at Philadelphia, and while the results which he reported were very fine, he was yet to be convinced that equally good ones were not to be obtained in other ways. One thought had struck him in reference to the inutility of operating under a continuous spray. For instance, a patient is brought into a hospital for an amputation, the operation and subsequent dressings are all made under a spray of carbolized water, with a good result as regards union by first intention, etc. Another patient is brought in with a bad compound fracture, received perhaps in some locality where the contact of disease germs with the wound would almost be a matter of course. Some hours elapse between the receipt of the injury and the application of the dressings, ample time being given for the infection of the wound. The first and subsequent dressings are made under carbolized spray, and a union without suppuration is the result. Now the query is, if the employment of the spray, or thorough washing with carbolized water is sufficient to destroy the germs which have already gained access to this wound, why will not the same method be sufficient in destroying the activity of the few germs which gain access to the stump during the brief process of amputation, without resorting to the troublesome method of operating under a spray.

Some discussion followed on the method employed by Lister in making his dressings, and after the appointment of Dr. Abbott as essayist, with Dr. Cronyn as alternate, the Association adjourned.

